# ABO-incompatible kidney transplantation in elderly patients over 60 years of age

**DOI:** 10.1007/s11255-012-0231-z

**Published:** 2012-07-25

**Authors:** Junji Uchida, Tomoaki Iwai, Yuichi Machida, Nobuyuki Kuwabara, Kazuya Kabei, Masaki Murao, Taiyo Otoshi, Toshihide Naganuma, Norihiko Kumada, Tatsuya Nakatani

**Affiliations:** Department of Urology, Osaka City University Graduate School of Medicine, 1-4-3, Asahi-machi, Abeno-ku, Osaka, 545-8585 Japan

**Keywords:** Kidney transplantation, ABO-incompatible, Elderly patients, Desensitization protocol

## Abstract

**Introduction:**

Patients aged 60 years and older represent the fastest-growing population with end-stage renal disease worldwide, and the need for a kidney transplant among this population is increasing. Due to the severe shortage of deceased donors in Japan, ABO-incompatible living donor kidney transplantation has been performed since the late 1980s. Excellent long-term outcomes have been achieved, and the rates of graft survival in these patients are currently similar to those in recipients of ABO-compatible grafts. However, the outcomes of ABO-incompatible kidney transplantation in elderly patients over 60 years of age have not been well studied yet.

**Patients and methods:**

We studied 4 elderly kidney transplant patients who received their grafts from ABO-incompatible living donors at our institution between December 2006 and December 2011, focusing on the immunosuppressive protocols, complications and graft survivals. The mean observation period was 21.5 months (range, 8 months to 62 months). Our immunosuppressive protocols were as follows: to remove the anti-A/B antibodies, the patients underwent 4–8 sessions of double-filtration plasmapheresis and/or plasma exchange prior to kidney transplantation until the anti-A/B titers were less than 1:16. For the patients with low anti-A/B titers (<1:512), the immunosuppressive protocol consisted of a single dose of rituximab (150 mg/m^2^). The patients with high anti-A/B antibody titers (≥1:512) underwent splenectomy and received 2 doses of rituximab. The pretransplant immunosuppressive protocol included B-lymphocyte suppression with 4 weeks of mycophenolate mofetil (0.5 g/day for low-titer protocol and 1 g/day for high-titer protocol).

**Results:**

All 4 patients underwent successful transplantation. At the end of follow-up, their mean serum creatinine was 1.18 mg/dl. No patient experienced antibody-mediated rejection or acute cellular rejection. Late-onset neutropenia occurred in two cases. Two cases experienced cytomegalovirus reactivation by cytomegalovirus antigenemia. In one patient, diffuse hemorrhage required surgical intervention. However, there were no severe complications.

**Conclusions:**

Although a careful evaluation of patients is needed, ABO-incompatible kidney transplantation may become a viable treatment option for elderly patients with end-stage renal disease.

## Introduction

Patients aged 60 years and older represent the fastest-growing population with end-stage renal disease worldwide [1], and the median age of end-stage renal disease (ESRD) stands at 66.2 years of age according to the 2010 data for Japan. As a result, a more specific strategy is needed for ESRD care in patients over 60 years. Mortality rates for individuals on dialysis 65 years of age and older are nearly seven times higher than those for the general population [2]. Kidney transplantation is the preferred treatment for ESRD, because it has been found to be associated with greater longevity and better quality of life compared with dialysis [3]. Previous studies have shown a significantly longer life expectancy among patients aged 60 years and older with deceased donor kidney transplantation compared with patients aged 60 years and older on the waiting list [4]. Due to the severe shortage of deceased donors in Japan, ABO-incompatible living donor kidney transplantation has been performed since the late 1980 s. Excellent long-term outcomes have been achieved, and the rates of graft survival in these patients are currently similar to those in recipients of ABO-compatible grafts [5]. However, the outcomes of ABO-incompatible kidney transplantation in elderly patients older than 60 years of age have not been well studied yet. Because ABO-incompatible kidney transplantation is immunologically a high-risk procedure, standard immunosuppressive regimens for ABO-incompatible kidney transplantation may elevate the risk of infectious complications in older recipients with a less active immune system. In this study, we demonstrated our modifications in immunosuppressive protocols as well as complications and graft survivals in elderly recipients of ABO-incompatible kidney transplants older than 60 years of age.

## Patients and methods

Four patients aged 60 years and older who received their grafts from ABO-incompatible living donors at our institution between December 2006 and December 2011 were enrolled in this study. The patients had anti-A/B titers ranging from 1:16–1:2,048. The donors were spouses (*n* = 3) and a child (daughter) (*n* = 1). The patient characteristics are shown in Table 1. All patients were followed until February 2012. The mean observation period was 1 year and 9.5 months (range, 8 months to 5 years and 2 months). The causes of ESRD were chronic glomerulonephritis (*n* = 1), renal sclerosis (*n* = 1) and unknown (*n* = 2). The cytomegalovirus serostatus pattern was donor positive/recipient positive in all cases.

**Table 1:** Patient characteristics

Patient	Gender (M/F)	Age (year)	Cause of ESRD	HD duration (month)	Donor	Donor age (year)	Preoperative donor S–Cr (mg/dl)	Preoperative donor eGFR (ml/min/1.73 m^2^)	HLA mm	Blood incompatibility		CNI
1	M	64	CGN	77	Wife	62	0.60	76.7	5/6	B	A	Tac
2	M	74	Renal sclerosis	8	Daughter	50	0.60	81.6	3/6	AB	B	CsA
3	M	70	Unknown	40	Wife	65	0.50	92.4	4/6	A	O	CsA
4	M	69	Unknown	50	Wife	66	0.60	75.3	3/6	B	A	CsA

*ESRD* end-stage renal disease, *HD* hemodialysis, *S*–*Cr* serum creatinine, *eGFR* estimated glomerular filtration rate, *HLA* human leukocyte antigen, *mm* mismatch, *CGN* chronic glomerulonephritis, *CNI* calcineurin inhibitor, *Tac* tacrolimus, *CsA* cyclosporin

## Immunosuppressive protocols

**Fig. 1:**
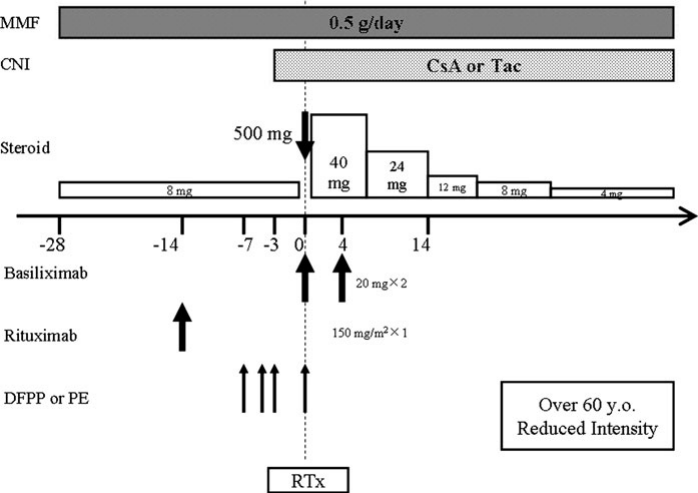
Immunosuppressive protocol for ABO-incompatible kidney transplantation without splenectomy in elderly recipients. *Tx* kidney transplantation, *MMF* mycophenolate mofetil, *PE* plasma exchange, *DFPP* double-filtration plasmapheresis

**Fig. 2:**
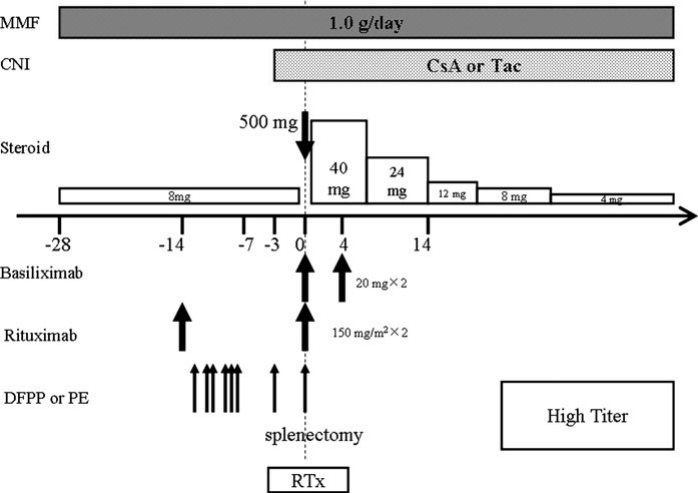
Immunosuppressive protocol for ABO-incompatible high-titer kidney transplantation. *Tx* kidney transplantation, *MMF* mycophenolate mofetil, *PE* plasma exchange, *DFPP* double-filtration plasmapheresis, *SPX* splenectomy

Previously, we reported on our standard desensitization protocol for ABO-incompatible kidney transplantation using rituximab without splenectomy at our institution. Briefly, this standard desensitization protocol consisted of 2 doses of rituximab 150 mg/m^2^ at 2 weeks prior to and on the day of transplantation, and pretransplant immunosuppression included B-lymphocyte suppression with 4 weeks of mycophenolate mofetil (MMF) 1.0 g/day and methylprednisolone (MP) 8 mg/day [6, 7]. This protocol was modified for our patient group aged 60 years and older. The recipients with low anti-A/B titers received a desensitization protocol without splenectomy consisting of only a single dose of rituximab 150 mg/m^2^ at 2 weeks prior to the transplantation, and pretransplant immunosuppression included B-lymphocyte suppression for 4 weeks of MMF 0.5 g/day and MP 8 mg/day to avoid over-immunosuppression (Fig. 1). The recipient with high anti-A/B titers received a desensitization protocol consisting of 2 doses of rituximab 150 mg/m^2^ at 2 weeks prior to and on the day of transplantation, splenectomy and pretransplant administration of 4 weeks of MMF 1 g/day and MP 8 mg/day (Fig. 2) [6–9].

To remove the anti-A/B antibodies, the three recipients with low anti-A/B titers (<1:512) underwent standard antibody removal consisting of 3 sessions of double-filtration plasmapheresis (DFPP) and 1 session of plasma exchange (PE). When the anti-A/B antibody titers did not decrease to less than 1:16, additional antibody removal was performed. The one recipient with high titers (≥1:512) underwent 5 sessions of DFPP and 3 sessions of PE prior to kidney transplantation until the anti-A/B titers were ≤1:16.

For postoperative immunosuppression, the same regimen as that for ABO-compatible cases was followed, in which calcineurin inhibitors were initiated 3 days before transplantation combined with two doses of basiliximab. Cyclosporin was given so as to maintain a blood trough level of 250–300 ng/ml during the first month after operation, 200–250 ng/ml during the second month, 150–200 ng/ml during the third month and 100–150 ng/ml thereafter. Tacrolimus was given so as to maintain a blood trough level of 10–13 ng/ml during the first month after operation, 8–10 ng/ml during the second month, 6–8 ng/ml during the third month and 3–6 ng/ml thereafter. Basiliximab was infused on day 0 and 4 at a dose of 20 mg. The MMF dosage after transplantation was maintained at pretransplant doses in both protocols. These protocols were approved by our Human Ethics Committee. All subjects gave informed consent for participation in the study. All procedures were in accordance with the Helsinki Declaration of 2000.

Anti-A/B antibody titers were measured pre- and post-transplantation. The anti-IgM titer was measured using the saline agglutination technique, and anti-IgG titer was measured using the indirect Coombs’ test.

Surveillance biopsies were performed once within a month after surgery and before discharged from hospital in all patients. When clinically indicated by rising serum creatinine or decreasing urine output, episode biopsies were performed.

## Results

All 4 patients had immediate graft function and underwent successful kidney transplantations. In all cases, no apparent cellular or humoral rejection was observed during the observation periods after kidney transplantation. None of the cases received episode biopsies. Subclinical rejection was not diagnosed on surveillance biopsies in any of the recipients. All recipients had good graft function (Table 2).

**Table 2:** Outcomes and complications

Patient	Acute cellular rejection	Antibody- mediated rejection	Graft survival	Follow-up (month)	Current S–Cr (mg/dl)	Current eGFR (ml/min/1.73 m^2^)	CMV antigenemia	Other complications
1	–	–	Yes	62	1.24	45.5	+	–
2	–	–	Yes	16	0.88	64.4	+	Postoperative hemorrhage
3	–	–	Yes	8	1.35	41.3	–	Pneumocystis pneumonia
4	–	–	Yes	8	1.25	44.9	–	–

*S*–*Cr* serum creatinine, *eGFR* estimated glomerular filtration rate, *CMV* cytomegalovirus

### Clinical course of patients (Table 2)

#### Patient 1

A 64-year-old man with end-stage chronic renal failure due to chronic glomerulonephritis visited our hospital to receive ABO-incompatible (type B to A) living-unrelated donor kidney transplant from his 62-year-old wife. The anti-B antibody titer was 1:2,048 using the Coombs’ method. Because of a high (more than 1:512) anti-A antibody titer, the patient underwent both administration of rituximab and splenectomy as desensitization. Immunosuppressive therapy consisted of tacrolimus, MMF, basiliximab and methylprednisolone. The kidney transplantation was performed uneventfully. He became positive for cytomegalovirus antigenemia 48 days after transplantation. However, no obvious invasive tissue disease occurred. As preemptive therapy, a 10-day administration of ganciclovir induced the elimination of cytomegalovirus antigenemia.

#### Patient 2

A 74-year-old man with end-stage chronic renal failure due to renal sclerosis visited our hospital to receive ABO-incompatible (type AB to B) living-related donor kidney transplant from his 50-year-old daughter. The patient received only a single dose of rituximab without splenectomy as desensitization. The kidney transplantation was performed uneventfully. However, on postoperative day 1 urine output decreased suddenly, and he was suffered from abdominal fullness. Computer tomography showed postoperative diffuse hemorrhage in pelvis, leading to surgical intervention. Thereafter, he was uneventful. Immunosuppressive therapy consisted of cyclosporin, MMF, basiliximab and methylprednisolone. However, he became positive for cytomegalovirus antigenemia 24 days after transplantation. Although ganciclovir was administered for 12 days, the patient did not become negative for cytomegalovirus antigenemia and experienced ganciclovir-induced leukocytopenia. After the discontinuation of ganciclovir, this patient was successfully treated with foscarnet. He experienced late-onset neutropenia 116 days after transplantation due to the administration of rituximab and MMF. Other drugs that could induce myelosuppression were not given to these patients at neutropenia onset, and the primary causes of neutropenia such as viral and bacterial infections did not occur. He recovered from neutropenia by granulocyte colony-stimulating factor administration.

#### Patient 3

A 70-year-old man with end-stage chronic renal failure visited our hospital to receive ABO-incompatible (type A to O) living-unrelated donor kidney transplant from his 65-year-old wife. The patient received only a single dose of rituximab without splenectomy as desensitization. Immunosuppressive therapy consisted of cyclosporin, MMF, basiliximab and methylprednisolone. The kidney transplantation was performed uneventfully. Since the first two patients developed cytomegalovirus viremia, a prophylaxis protocol for cytomegalovirus was applied to this patient. He had an elevation of C-reactive protein and β-D-glucan 80 days after kidney transplantation. Because computer tomography of the chest showed interstitial pneumonitis, pneumocystis pneumonia was suspected. Interstitial pneumonitis was treated by administration of sulfamethoxazole trimethoprim. He experienced neutropenia 95 days after transplantation. He recovered from neutropenia by granulocyte colony-stimulating factor administration.

#### Patient 4

A 69-year-old man with end-stage chronic renal failure due to chronic glomerulonephritis visited our hospital to receive ABO-incompatible (type B to A) living-unrelated donor kidney transplant from his 66-year-old wife. The patient received only a single dose of rituximab without splenectomy as desensitization. Immunosuppressive therapy consisted of cyclosporin, MMF, basiliximab and methylprednisolone. The kidney transplantation was performed uneventfully. A prophylaxis protocol for cytomegalovirus was also applied to this patient.

### Changes in ABO antibody titers

Although patient 1 with high anti-A/B titers experienced antibody rebound slightly after plasmapheresis, the rebound was eliminated after the third or fourth plasmapheresis. The other cases with low titers did not experience any rebound of ABO antibody titers (Table 3).

**Table 3:** Anti-A/B antibody titers

Patient	Initial antibody titer		Preoperative antibody titer	
	IgG	IgM	IgG	IgM
1	×2,048	×512	×2	×2
2	×2	×2	×1	×1
3	×256	×8	×16	×1
4	×32	×8	×4	×2

## Discussion

The outcomes of ABO-incompatible kidney transplantation in elderly patients older than 60 years of age have not been well studied yet. The present study demonstrated our modifications in immunosuppressive protocols for these recipients. All of our patients underwent successful transplantations with no severe complication or rejection. To our knowledge, this is the first demonstration of ABO-incompatible kidney transplantation in elderly patients over 60 years. Our results suggested that this method is a radical but effective treatment for elderly patients with ESRD.

Transplantation improves the cumulative survival of ESRD patients compared with dialysis as shown by Wolfe et al. [10]: Individuals 60–74 years of age who underwent transplantation showed an improvement in cumulative 1-year post-transplantation survival rate compared with those only listed for transplantation. They revealed an increased 4-year projected life span and a 61 % decrease in the long-term risk of death and also showed that mortality rates of 23.2 deaths per 100 patient-years if left on dialysis decreased to 7.4 deaths per 100 patient-years following transplantation. It has also been reported that although the survival benefit of transplantation decreases as the wait time increases, elderly patients continue to derive a survival benefit if they undergo transplantation within 3 years of dialysis therapy initiation [11]. Moreover, older recipients have been reported to demonstrate improved patient and graft survivals following transplantation from living donors [12]. During the early phase of dialysis therapy or as preemptive kidney transplantation, ABO-incompatible kidney transplantation from living donors may be worthwhile for patients aged 60 years and older, considering the survival benefit.

In 3 out of the 4 recipients, the donors were spouses aged 60 years and over, and their graft function was satisfactory (mean S–Cr, 1.28 mg/dl; mean eGFR, 43.8 ml/min/1.73 m^2^). A previous report demonstrated excellent graft and patient survival in elderly recipients from older living donors, showing that older living donors may be an important option for elderly transplantation candidates and should be considered for older patients with a willing and suitable older donor [13]. ABO-incompatible spousal kidney transplantation could therefore provide a successful alternative option for elderly patients with ESRD.

Beyond increases in life expectancies, previous reports have revealed that elderly recipients (>60 years and older) of kidney transplants have improved quality of life including physical functioning and mental health on nationally standardized SF-36 questionnaires. These results were found to be equivalent to national age and sex appropriate norms. It should be appreciated that these results were reported despite a significantly reported incidence of surgical and post-transplant medical complications [14]. All elderly patients in our study underwent successful transplantations with no severe complications. These results indicated that ABO-incompatible kidney transplantation may improve the quality of life of elderly patients with ESRD.

Although ABO-incompatible kidney transplantation is an immunologically high-risk procedure, the development of powerful immunosuppressant drug combinations has resulted in excellent graft and patient survivals [5]. Increasing age is associated with structural and functional changes in body compartments and tissues that alter absorptive capacity, volume of distribution, hepatic metabolic function and ultimately drug disposition. Age-related changes occur in most organs and can alter pharmacodynamic responses to medications. These alterations could potentially affect drug responsiveness and result in altered toxicity or influence drug–drug interactions [15]. Although no data have been published on these subjects, elderly patients who have received ABO-incompatible kidney transplantation are more susceptible to developing adverse effects related to immunosuppressant therapy. In our present study, we modified our desensitization protocol in order to guarantee better short- and long-term efficacy and minimize toxicity. The standard desensitization protocol at our institution for ABO-incompatible kidney transplantation (younger than 60 years of age, lower than 1:512 of anti-A/B titers) consisted of 2 doses of rituximab 150 mg/m^2^ at 2 weeks prior to transplantation, and pretransplant immunosuppression included B-lymphocyte suppression with 4 weeks of MMF 1.0 g/day and MP 8 mg/day [6, 7]. The desensitization protocol modified for elderly recipients consisted of only a single dose of rituximab 150 mg/m^2^ at 2 weeks prior to transplantation, and pretransplant immunosuppression included B-lymphocyte suppression with 4 weeks of MMF 0.5 g/day and MP 8 mg/day to avoid over-immunosuppression. In our case series, there were no cytomegalovirus serology–naïve kidney transplant patients of cytomegalovirus serology–positive organs. However, intractable cytomegalovirus reactivation occurred in one case, and thereafter, measures were taken for prophylaxis of cytomegalovirus. One patient experienced an elevation of β-d-glucan, for which *Pneumocystis jiroveci* infection was suspected. Two patients experienced late-onset neutropenia. Although spousal kidney transplantation, which could have presented poor tissue antigen compatibility, was performed in 3 of the 4 cases, there were no episodes of acute cellular or humoral rejection during the follow-up period. Furthermore, none of the patients experienced any rebound of ABO antibody titers during the pre- and postoperative period. Although our modified protocol for elderly recipients had enough immunosuppressive effectiveness to control the elevation of antibody titers and acute rejection, it may induce over-immunosuppression. An effort to develop a tailored desensitization protocol for elderly recipients of ABO-incompatible kidney transplants may be necessary.

Recipients with high antibody titers at baseline have been reported to be at high risk of antibody-mediated rejection and graft loss [16]. ABO-incompatible high-titer kidney transplantation has remained a medical challenge. Even if the recipients with high titers are older than 60 years of age, their ability to produce antibodies and their immune response against grafts may be high. Previously, we demonstrated a desensitization protocol for ABO-incompatible high-titer kidney transplantation consisting of rituximab infusion, splenectomy, plasmapheresis and pharmacological immunosuppression [8, 9]. We applied this desensitization protocol to the case with high antibody titers, and successful transplantation was accomplished in this patient with no severe complications.

In conclusion, this small preliminary study showed that ABO-incompatible kidney transplantation could be accomplished in elderly recipients without any severe complications. Although complications including cytomegalovirus reactivation, diffuse hemorrhage and pneumocystis pneumonia occurred, all patients recovered successfully. Elderly patients receiving ABO-incompatible kidney transplantation are more susceptible to developing adverse effects related to immunosuppressant therapy. An effort to develop a tailored protocol for elderly recipients of ABO-incompatible kidney transplants may be necessary. Although a careful evaluation of the comorbidities of patients is needed, ABO-incompatible kidney transplantation may become a viable treatment option for elderly patients with ESRD. Larger and longer trials are further needed to assess the safety and effectiveness of this procedure.
